# Body mass index modifies the association between occupational noise exposure duration and ultrasound‑detected hepatic steatosis: A cross-sectional study among male factory workers

**DOI:** 10.1371/journal.pone.0358275

**Published:** 2026-09-11

**Authors:** Dandan Liu, Huixia Ji, Ye Chen, Tongzhou Zhou

**Affiliations:** 1 Department of Occupational Disease, Nanjing Prevention and Treatment Center for Occupational Diseases, Nanjing, Jiangsu, China; 2 Clinical Laboratory, Nanjing Prevention and Treatment Center for Occupational Diseases, Nanjing, China; University of Diyala College of Medicine, IRAQ

## Abstract

**Background:**

Metabolic dysfunction‑associated steatotic liver disease (MASLD) is a multisystem metabolic disorder. The impact of occupational noise exposure on metabolic processes through non-auditory pathways is also closely associated with hepatic steatosis in addition to obesity. Most studies examine noise exposure or Body Mass Index (BMI) alone on ultrasound-detected hepatic steatosis(UDHS), while few examine BMI’s interaction with occupational noise duration. The underlying mechanisms of these combined effects remain unclear. The correlation between occupational noise exposure duration and UDHS in automotive manufacturing workers is examined, as well as the interactive role of BMI

**Objective:**

This study investigates the correlation between occupational noise exposure duration and UDHS in automotive manufacturing workers, as well as the interactive moderating effect of BMI on this association.

**Methods:**

A retrospective study involved 769 male workers exposed to occupational noise. Ultrasound was used to diagnose hepatic steatosis. Modified Poisson regression with robust variance estimation was used to analyze variable associations. A slope analysis and Johnson-Neyman test were used to validate BMI and variable interactions. A sensitivity analysis was performed to ensure robustness.

**Results:**

Overall, 53.2% of workers had UDHS. Noise exposure duration showed a significant independent association with UDHS,with a negative interaction effect on BMI (P = 0.006) after adjusting for confounding factors. In normal-weight workers, each additional year of noise exposure was associated with an 8.2% higher UDHS likelihood (P < 0.001), but not statistically significant in overweight/obese individuals; BMI = 27.7 kg/m² represented the critical threshold where the magnitude of this association approached zero. Alcohol consumption and overweight/obesity were independent risk factors.

**Conclusion:**

Noise exposure is associated with UDHS risk among male workers, yet BMI modifies this association, whereby the magnitude of this association weakens as BMI increases. These findings are specific to male factory workers and cannot be generalized to female populations. Accordingly, tailored occupational health protection strategies can be developed for noise-exposed populations.

## Introduction

Metabolic dysfunction-associated steatotic liver disease (MASLD) is defined as hepatic steatosis confirmed by imaging or histology, accompanied by at least one of the following five cardiovascular and metabolic risk factors: overweight/obesity, type 2 diabetes mellitus/prediabetes, hypertension, hypertriglyceridemia, or low high-density lipoprotein cholesterol(LDL-C) [1]. Compared to the previous definition of “non-alcoholic fatty liver disease (NAFLD),” MASLD places greater emphasis on the role of metabolic risk factors in the progression of hepatic steatosis. MASLD, the most prevalent chronic liver disorder globally, affects approximately 32.4% of the population and exhibits a steadily increasing prevalence [2]. Body Mass Index (BMI), as the core index to measure the degree of obesity, has a clear correlation with the occurrence and development of fatty liver, especially with MASLD. Epidemiological studies have confirmed that obesity is considered to be a key factor in the etiology, pathogenesis and outcome of MASLD [3]. Epidemiological evidence consistently identifies substantial heterogeneity in MASLD prevalence across different BMI categories. Among men, MASLD prevalence was markedly higher in those with a BMI ≥ 30 kg/m² [4]. TyG-BMI and other BMI-related indicators have excellent predictive ability for the incidence of MASLD [5]. A study found that individuals with low BMI coupled with elevated WC-related indices exhibited the highest mortality risk [6]. Genetic association study also found that parents with overweight or obesity were 3.73-fold more likely to have MASLD (OR 3.73, 95% CI 2.43 - 5.73) than parents with normal BMI, and 67% of the association was mediated by children’s cumulative excess BMI [7]. Collectively, existing research indicates that the role of BMI in the prognostic assessment of MASLD may be more complex than conventional understanding.

Occupational noise exposure—recognized as a widespread occupational hazard in automotive manufacturing, mechanical processing, mining, and related sectors—exerts systemic physiological effects beyond auditory impairment. Animal experiments have shown that long-term low-intensity noise exposure (75 dB SPL for 3 months) can aggravate the progression of NAFLD induced by high-fat diet in mice, which is manifested as hepatocyte steatosis, lipid metabolism disorder and increased inflammation. Further mechanism study revealed that the plasma adrenocorticotropic hormone (ACTH) level in the noise-exposure group was 1.5 times higher than that in the high-fat diet group, and the activation of hypothalamic-pituitary-adrenal (HPA) axis played a key role in this increase [8]. Study found an association between occupational noise exposure and metabolic syndrome (MetS) and its components, and a positive association between cumulative noise exposure and MetS risk [9]. The association between long-term noise exposure and increased adipose tissue distribution and liver fat content further supports the potential effect of noise exposure on metabolic function. The findings indicate that noise‑exposure‑related metabolic disturbances may exacerbate deviations in BMI, while both environmental noise exposure and BMI abnormalities are independently associated with hepatic steatosis. The prevalence of MASLD was different in different BMI categories [4,10]. Therefore, BMI may serve as a critical effect modifier in the association between noise exposure and MASLD. Yet, most epidemiological studies have treated BMI solely as a confounding variable in statistical models, with limited investigation into the synergistic or interactive effects of BMI and cumulative noise exposure duration on MASLD risk. Moreover, the potential moderating role of BMI—particularly in relation to weight status—remains uncharacterized.

The study analyzes the correlation between noise exposure duration and ultrasound‑detected hepatic steatosis (UDHS) for workers in a large-scale automotive manufacturing plant. This study examines the interaction effect between BMI and noise exposure duration and performs a sensitivity analysis. By providing a scientific basis for comprehensive prevention and control of hepatic steatosis among occupational noise-exposed population, this study will also promote the development of targeted occupational health interventions, enhancing the research framework for occupational noise‑related non-auditory system damage.

## Methods

### Study design and population

The study recruited workers from a local automobile manufacturing plant who underwent occupational health examinations in 2025. To ensure adequate cumulative noise exposure, workers must have worked at the plant for at least one year. The exclusion criteria included: (1) Individuals who declined blood sampling on the day of the examination; (2) Workers who had a pre-occupational history of hyperlipidemia; (3) Female workers excluded because the sample size was insufficient(n < 10); and (4) Male workers who consumed more alcohol than 30 grams a day. A total of 769 workers were studied. Nanjing Institute for Occupational Disease Prevention Ethics Committee approved the study protocol (Approval No.: 2025–005), and all procedures were performed in accordance with the Declaration of Helsinki. As this was a retrospective study utilizing existing occupational health examination data and occupational hazard records provided by the employer, written consent was waived. Data for this study was accessed on 1 March 2026. Participants’ identifiable information was available during and after data collection, and all data were anonymized prior to analysis.

### Data collection and variable definition

Data was collected on demographics, past smoking habits, alcohol consumption, medical history, chronic disease medications, BMI, and other relevant information. Workers’ occupational noise exposure history was provided by their employers, with only the duration of exposure documented. After fasting, subjects underwent ultrasonography examinations in the early morning. Liver ultrasound demonstrated mild, moderate, or severe ultrasound‑detected hepatic steatosis. Ultrasound‑detected hepatic steatosis: Abdominal US assessed liver echogenicity relative to the kidney, portal vein wall, and diaphragm to grade the severity of hepatic steatosis [11]. Affiniti 70 color Doppler ultrasound scanner (Philips, America) with 3.5 MHz probe frequency was used. BMI was calculated as the body weight (kg) divided by the square of height (kg/m^2^). Smoking (including current and ever smoking) was defined as smoking three cigarettes per day or more for at least half a year. Although the threshold of ≥3 cigarettes per day is less commonly adopted than the conventional cutoff of ≥1 cigarette per day, accumulating epidemiological evidence indicates that even light daily smoking (1–4 cigarettes per day) is associated with significantly elevated risks of chronic disease [12]. Specifically, low-intensity smoking (2–5 cigarettes per day) has been linked to a 50% higher risk of cardiovascular disease and a 60% higher risk of all-cause mortality compared with never smoking [13].We selected the threshold of ≥3 cigarettes per day to more precisely identify individuals with sustained, regular tobacco exposure while excluding sporadic or episodic smoking behaviors. Alcohol consumption was defined as weekly drinking for at least one year.

### Statistical analyses

#### The normality of continuous variables was automatically evaluated via the Shapiro – Wilk test.

Continuous variables with a normal distribution are presented as mean ± standard deviation, and non-normal variables as median (interquartile range). Independent samples t-tests and Mann-Whitney U tests were used for comparisons between groups. The chi – squared test was used to assess intergroup differences in categorical variables. Baseline characteristics were compared and described among different BMI groups.

#### Multivariate principal analysis.

Due to the high incidence of UDHS in this cohort (>10%), traditional logistic regression would overestimate the effect size. In order to estimate the prevalence ratio and its 95% confidence interval, we used modified Poisson regression coupled with robust variance estimation (sandwich estimator, Huber-White HC0 type). During the study, the discrete parameters of the Poisson model were evaluated, further emphasizing the need for robust standard errors. The multivariate model adjusted for age, smoking, and alcohol consumption.

#### Interaction effects and simple slope analysis.

To assess whether BMI moderates the association between noise exposure duration and UDHS, an interaction term between noise exposure duration and continuous BMI was incorporated into the regression model. If the interaction reached statistical significance, simple-slope decomposition was performed. Marginal effects (β-coefficients) and corresponding standard errors (SE) for noise exposure duration were computed for illustrative subgroups corresponding to normal-weight (BMI < 24 kg/m²) and overweight/obese (BMI ≥ 24 kg/m²) ranges. These subgroup estimates were derived post-hoc from the continuous-BMI model, rather than fitting a separate categorical-BMI regression. Standard errors for marginal effects were calculated via the Delta method, fully accounting for covariance between coefficients of main terms and the interaction term.

#### Sensitivity analysis and the Johnson-Neyman technique.

An exploratory regression model was fitted with binary BMI (BMI < 24 kg/m² vs. BMI ≥ 24 kg/m²) to examine whether the interaction pattern observed in the primary continuous‑BMI model was robust. This categorical classification was based on the optimal population‑specific cutoff for Chinese adults, which is more sensitive for identifying metabolic risks than the conventional WHO 25 kg/m² threshold [14,15]. In addition, the Johnson-Neyman technique was applied within the primary continuous-BMI interaction model to identify the exact continuous-BMI threshold above which the marginal association of noise exposure duration with UDHS became statistically non-significant. (i.e., the BMI value corresponding to a marginal prevalence ratio (PR) of 1.00). To visually depict disparities in absolute risk, logistic regression was used to generate predictive probability curves, ensuring that the predicted probabilities remained within the range of 0–1. Statistical analyses were conducted using R software (version 4.3.3). P values less than 0.05 were considered statistically significant.

## Results

### Basic characteristics of the study subjects and the prevalence of UDHS

A total of 769 male workers were enrolled in this study. In total, there were 409 cases of UDHS, yielding a prevalence rate of 53.2%. The baseline characteristics are presented in Table 1. Neither age (39.9 years vs. 40 years, P = 0.928) nor duration of exposure (13.0 years vs. 14.0 years, P = 0.060) differed statistically significantly between the two groups. Alcohol consumption rate was significantly higher in the UDHS group (17.4% vs. 10.8%, P = 0.013), whereas smoking status was not significantly different (P = 0.149). In the UDHS group, there were 79.5% (325/409) overweight or obese individuals, compared to 36.7% (132/360) in the Non-UDHS group (P < 0.001).

**Table 1:** Basic Characteristics of Study Subjects.

Variable	Total	Non-UDHS group	UDHS group	p value
	N = 769	N = 360	N = 409	
**Age**	39.9 (6.11)	39.9 (6.07)	40.0 (6.14)	0.928
**Noise exposure duration**	14.0 [9.00;17.0]	13.0 [8.00;17.0]	14.0 [10.0;17.0]	0.06
**BMI**	24.7 (3.08)	23.1 (2.64)	26.1 (2.72)	<0.001
**Overweight/obese**				<0.001
**0**	312 (40.6%)	228 (63.3%)	84 (20.5%)	
**1**	457 (59.4%)	132 (36.7%)	325 (79.5%)	
**Smoking**				0.149
**0**	362 (47.1%)	159 (44.2%)	203 (49.6%)	
**1**	407 (52.9%)	201 (55.8%)	206 (50.4%)	
**Alcohol**				0.013
**0**	659 (85.7%)	321 (89.2%)	338 (82.6%)	
**1**	110 (14.3%)	39 (10.8%)	71 (17.4%)	

### Interaction between noise exposure duration and BMI on UDHS

#### Multicollinearity assessment.

As cumulative noise exposure duration is intrinsically related to age, this study assessed multicollinearity before interpreting the regression models. A Pearson correlation analysis showed a moderate positive correlation (r = 0.417, 95% CI: 0.357–0.474, P < 0.001). These bivariate correlations did not result in significant multicollinearity problems in multivariate models. In both the Logistic and modified Poisson main – effects models, the variance inflation factors (VIF) of all covariates (including age, exposure duration, BMI, smoking, and alcohol consumption) were within the range of 1.00 to 1.22. Due to the low VIF values (VIF > 5), multicollinearity interference with parameter estimation on the model was clearly excluded, demonstrating the stability and reliability of the regression coefficients.

In this research, the incidence rate of UDHS outcomes was comparatively high (>10%). Logistic regression typically has a higher OR than true relative risk (RR). For estimating the prevalence ratio, modified Poisson regression with robust variance estimation was used. As presented in Table 2, the interaction term between the duration of noise exposure and continuous BMI demonstrated a highly significant negative correlation (β = −0.005, PR = 0.995, P = 0.006), confirming the robustness of the interaction effect.

**Table 2:** Sensitivity analysis for the association between noise exposure, continuous BMI, and UDHS.

Variables	Beta	Robust SE	Z value	P value	PR	95% CI
**(Intercept)**	−5.87	0.7779	−7.55	0	0.003	0.001-0.013
**Age**	−0.0017	0.0056	−0.31	0.7596	0.998	0.987-1.009
**Noise exposure duration**	0.144	0.0514	2.8	0.0051	1.155	1.044-1.277
**BMI**	0.2024	0.0278	7.29	0	1.224	1.159-1.293
**Smoking**	−0.0388	0.0618	−0.63	0.5298	0.962	0.852-1.086
**Alcohol**	0.2528	0.0732	3.45	0.0006	1.288	1.116-1.486
**Noise exposure duration×BMI**	−0.0052	0.0019	−2.75	0.0059	0.995	0.991-0.999

Footnote: BMI was treated as a continuous variable in this analysis. P for interaction (exposure duration × continuous BMI) = 0.006.

#### Simple slope analysis.

To visualize the clinical significance of this continuous interaction effect, the study performed a simple slope analysis based on the model at three crucial clinical cutoff values for adult BMI in China (18.5, 24.0, and 28.0 kg/m^2^) (Table 3). Increasing BMI attenuated the promotional effect of noise exposure duration on UDHS risk. UDHS risk increased by 4.9% each additional year of exposure at the lower normal weight threshold (BMI = 18.5 kg/m^2^) (PR = 1.049, 95% CI: 1.014–1.085, P = 0.006). The risk increased by 2.0% at the overweight threshold (BMI = 24.0 kg/m^2^) (PR = 1.020, 95% CI: 1.003–1.037, P = 0.022) (Table 3). The effect of noise exposure vanished completely when BMI reached 28 kg/m^2^ (PR = 0.999, 95% CI: 0.986–1.011, P = 0.829). When BMI reaches approximately 27.7 kg/m^2^, the effect estimate for noise exposure duration approaches zero.

**Table 3:** Marginal effects of noise exposure duration at specific Chinese clinical BMI cut-off points (Continuous BMI model).

groups	Beta	SE	Z value	P value	PR (95% CI)
18.5(Normal weight lower bound)	0.0479	0.0173	2.77	0.0056	1.049(1.014-1.085)
24 (Overweight threshold)	0.0194	0.0084	2.3	0.0215	1.020(1.003-1.037)
28 (Obese threshold)	−0.0014	0.0064	−0.22	0.8292	0.999(0.986-1.011)

Footnote: The Johnson-Neyman technique identified that the effect of noise exposure duration became null (PR = 1.00) at a BMI of 27.7 kg/m².

**Fig 1:**
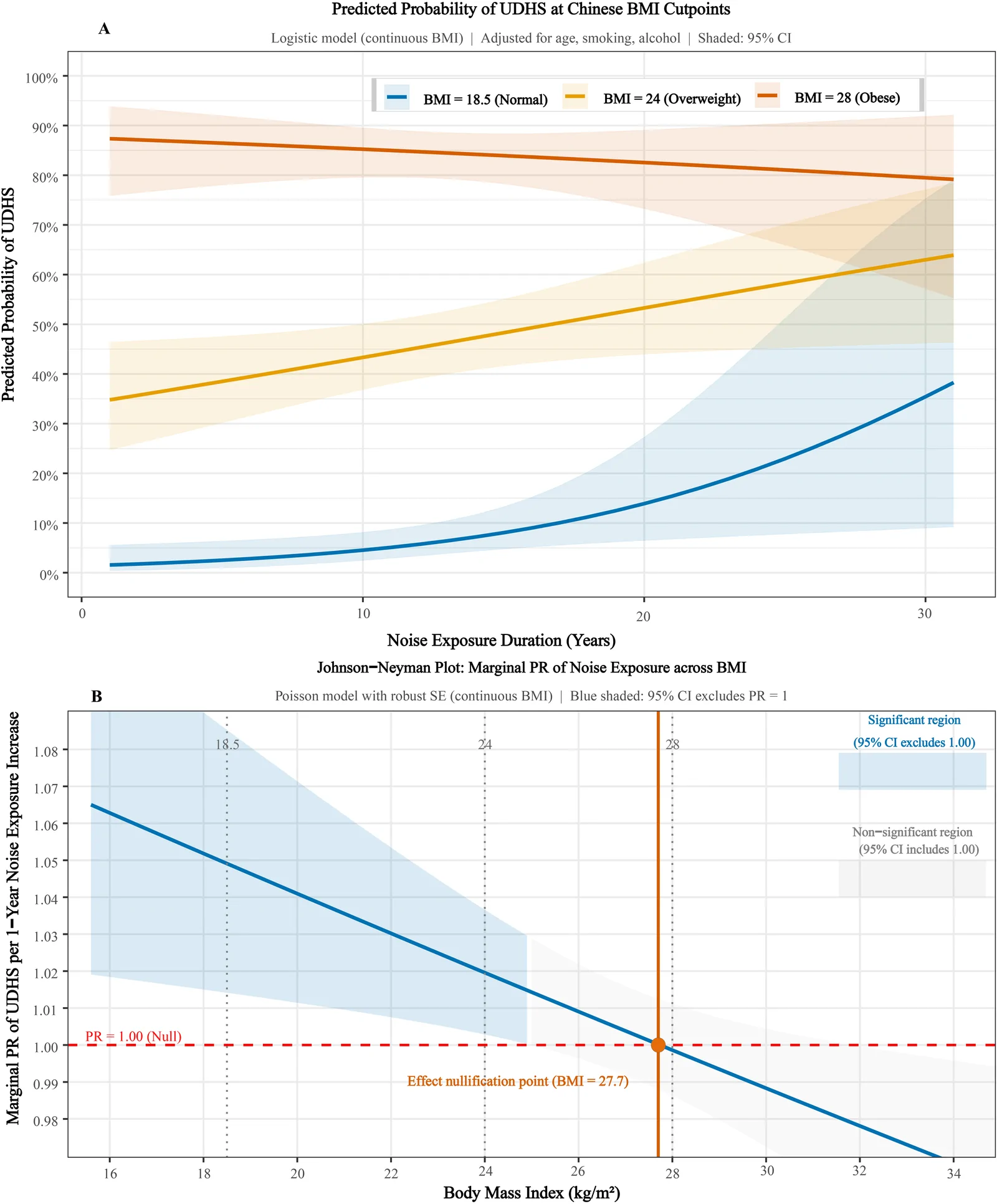
Visualizing the modifying effect of continuous BMI on the association between noise exposure duration and UDHS. A: Predicted probability curves of UDHS across noise exposure duration at three Chinese clinical BMI cut-offs (18.5, 24.0, and 28.0 kg/m²), Predictions were derived from a logistic regression model adjusted for age, smoking, and alcohol consumption. B: Johnson-Neyman plot illustrating the marginal PR of UDHS per 1-year increase in noise exposure across the continuous BMI spectrum (derived from a modified Poisson model with robust SEs).

Fig 1 shows the prediction probability trajectory and Johnson-Neyman interval plots based on the continuous BMI model in order to illustrate the continuous variation process. Fig 1A shows a significant gradient attenuation with increasing BMI cutoff values. The slope of the curve is steepest at the low end of normal weight (BMI = 18.5 kg/m^2^), decelerates at overweight (BMI = 24.0 kg/m^2^), and plateaus at obesity (BMI = 28.0 kg/m^2^). In Fig 1B, the blue solid line represents marginal PR, which intersects the null line exactly (PR = 1) on the left side. In the blue shaded area (i.e., the region where the lower limit of the 95% CI exceeds 1.00), noise exposure exerts statistically significant effects on BMI. At a BMI of approximately 27.7 kg/m^2^, this effect intersects the null line. When the BMI exceeds this critical point, the independent positive effect of noise exposure duration on UDHS risk loses statistical significance.

### Sensitivity analysis

#### Analysis of the interaction between noise exposure and BMI classification.

A significant interaction effect between noise exposure duration and BMI classification (normal weight vs. overweight/obese) was observed (PR = 0.924, 95% CI: 0.883–0.967, P = 0.0007) after adjusting for confounding factors (Table 4). Overweight/obesity was the strongest independent risk factor for UDHS. Compared with individuals of normal weight (BMI < 24 kg/m²), those with overweight/obesity (BMI ≥ 24 kg/m²) exhibited a significantly increased risk of UDHS by 7.558-fold (95% CI: 3.790–15.073, P < 0.001). After controlling for other variables, UDHS risk increased 8.2% with each additional year of exposure (PR = 1.082, 95% CI: 1.034–1.132, P = 0.0006). The risk of UDHS was also 1.318-fold higher for drinkers than non-drinkers (95% CI: 1.157–1.502, P < 0.001) (Table 4). A multivariate model showed no significant association between age (P = 0.424) and smoking (P = 0.242) with UDHS.

**Table 4:** Multivariate adjusted Poisson regression analysis of the association between years of noise exposure, BMI classification, and the interaction with UDHS.

Variables	Beta	Robust SE	Z value	P value	PR value	95% CI
**(Intercept)**	−2.1928	0.3741	−5.86	0	0.112	0.054-0.232
**Age**	−0.0044	0.0055	−0.8	0.4235	0.996	0.985-1.006
**Noise exposure duration**	0.079	0.0231	3.42	0.0006	1.082	1.034-1.132
**Overweight/obese**	2.0226	0.3522	5.74	0	7.558	3.790-15.073
**Smoking**	−0.0708	0.0605	−1.17	0.2418	0.932	0.827-1.049
**Alcohol**	0.2764	0.0666	4.15	0	1.318	1.157-1.502
**Noise exposure×overweight/obese**	−0.0791	0.0233	−3.4	0.0007	0.924	0.883-0.967

#### Marginal effect analysis across different BMI groups (simple slope analysis).

**Fig 2:**
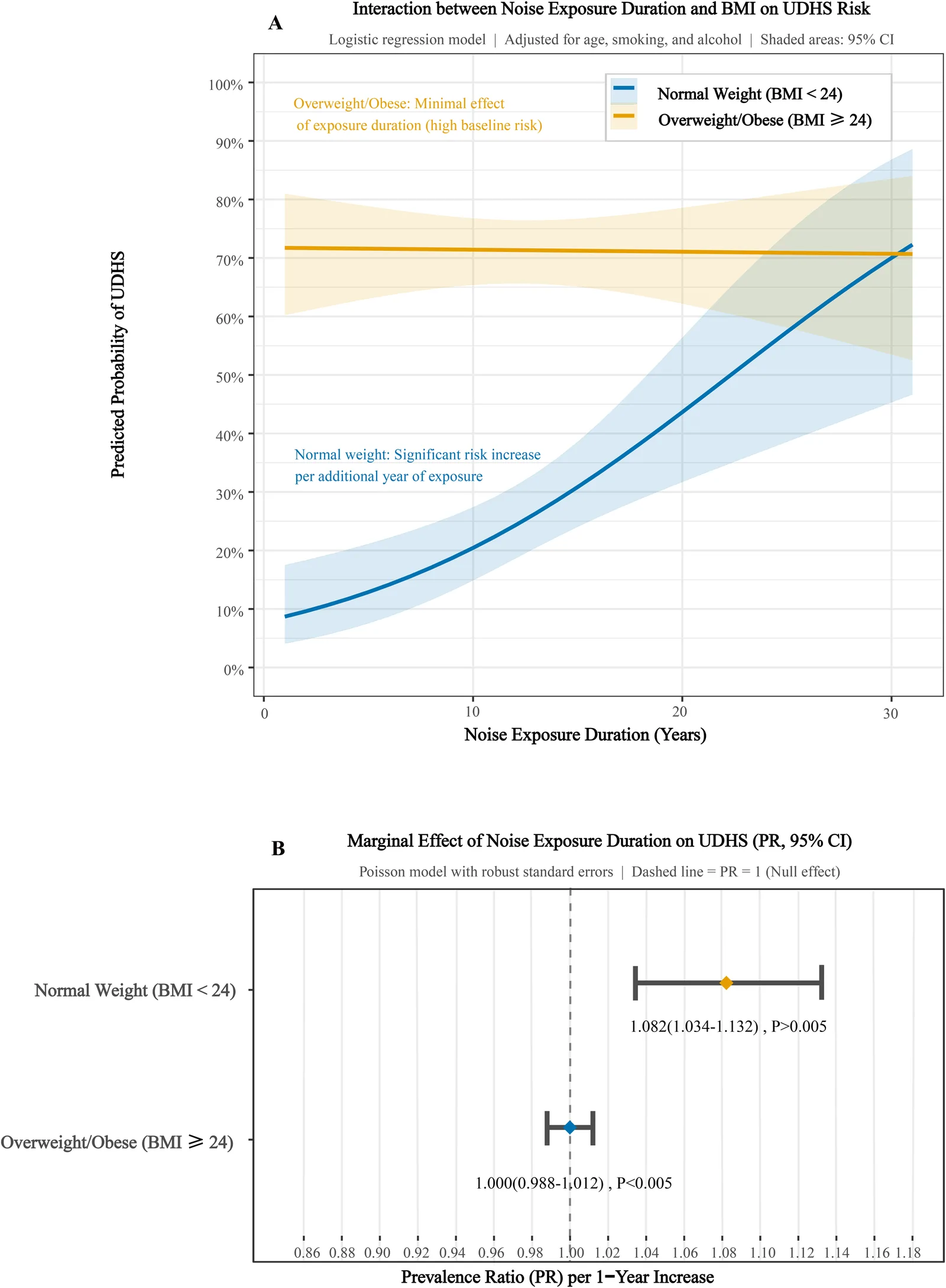
Diagram illustrating the modulatory effect of BMI on the association between noise exposure duration and UDHS A. Curve showing the predicted probability of UDHS stratified by BMI versus years of noise exposure. The normal group (BMI < 24 kg/m²) exhibits a steep upward trend, indicating that each additional year of exposure significantly increases the risk. In contrast, the baseline risk in the overweight/obese group (BMI ≥ 24 kg/m²) was already elevated, resulting in a flatter curve that reflects minimal marginal effects of increased exposure duration. B. Forest plot illustrating the marginal hazard ratio (HR) and 95% CI for each additional year of noise exposure regarding UDHS incidence. In the normal group, noise exposure was significantly associated with an increased risk of UDHS (HR = 1.082, 95% CI: 1.034–1.132, P < 0.05). In contrast, this association was entirely absent in the overweight/obese group (HR = 1.000, 95% CI: 0.988–1.012, P > 0.05). The dashed line represents the null hypothesis (HR = 1.00). The analysis was conducted using a modified Poisson regression model incorporating robust standard errors.

Simple slope analysis was used to quantify interaction effects. In individuals with normal BMI, each additional year of exposure increased the risk of UDHS by 8.2% (PR = 1.082, 95% CI:1.034–1.132, P < 0.001). In contrast, for overweight/obese individuals with BMI ≥ 24, noise exposure duration had no significant association with UDHS (PR = 1.000, 95% CI: 0.988–1.012, P = 0.987) (Table 5, Fig 2B).

**Table 5:** Stratified marginal effects of noise exposure duration on fatty liver by BMI category (Simple slope analysis).

groups	Beta	SE	Z value	P value	PR value	95% CI
**BMI < 24(normal)**	0.079	0.0231	3.42	0.0006	1.082	1.034-1.132
**BMI ≥ 24(overweight/obese)**	−0.0001	0.0061	−0.02	0.9869	1	0.988-1.012

Footnote: PR = Prevalence Ratio. Marginal effects were estimated from the modified Poisson regression models with robust standard errors, adjusting for age, smoking, and alcohol consumption. P for interaction (exposure duration × BMI category) = 0.001.

#### The predictive trend of disease prevalence probability.

Fig 2A visualizes the interaction phenomenon using a Logistic regression model. In individuals with normal body weight, the absolute prevalence probability of UDHS increases with increasing noise exposure. Among overweight/obese individuals, whose baseline risk is already extremely high (with a predicted probability approaching or surpassing 70%), extended noise exposure does not result in a further significant increase in prevalence. Increasing exposure duration results in distinct divergent curves.

## Discussion

Noise at work is a prevalent occupational hazard that not only is associated with hearing impairment over time but has also been linked to metabolic disorders and liver disease. Occupational noise exposure is associated with abnormal lipid metabolism and metabolic syndrome. Noise exposure and metabolic disruptions may be partially mediated by obesity [16,17]. Studies on animals indicate that chronic exposure to noise activates the HPA axis, facilitating the production of ACTH and glucocorticoids. The result is activation of hepatic glucocorticoid receptors and disruption of hepatic lipid metabolism [8]. Furthermore, noise regulates the microbiota and metabolites through the gut-liver axis, activating hepatic gluconeogenesis (CREB/CRTC2-PCK1) and lipid synthesis (SREBP1/SCD) pathways, thereby disrupting glucose and lipid metabolism [18].

In this research, among 769 male workers exposed to occupational noise, 409 (53.2%) had UDHS. A cross-sectional analysis of the U.S. population indicated an overall prevalence of hepatic steatosis of 21.1%, increasing from 18.6% in 2017 to 25.0% in 2023 (P = 0.092) [19]. In another Chilean study, 26.8% of the local population had hepatic steatosis [20]. Hepatic steatosis may be more prevalent in individuals exposed to occupational noise. The proportion of alcohol consumption was significantly higher in the UDHS group than in the Non-UDHS group, further confirming that alcohol intake is a major risk factor. Even moderate alcohol consumption can exacerbate hepatic steatosis through oxidative stress, lipid metabolic disorders, and gut microbiota dysbiosis [21].

Noise exposure is positively correlated with obesity indicators like BMI and waist circumference, with noise’s impact on lipid metabolism partially mediated by obesity indices. BMI mediates the relationship between noise exposure and total cholesterol/high-density lipoprotein cholesterol (TC/HDL-C), LDL-C/HDL-C, and dyslipidemia prevalence, with mediation proportions of 24.45%, 24.33%, and 21.51%, respectively [16]. Noise exposure and BMI have not been fully elucidated in the development of UDHS, and there is no definitive conclusion regarding whether the magnitude of the association for noise exposure varies based on BMI. The study found that BMI and years of noise exposure interacted significantly on UDHS incidence for 769 individuals exposed to occupational noise: Noise exposure duration showed a significant association among individuals with low BMI, however, this association was attenuated among individuals with high BMI.

This study found that occupational noise exposure is associated with UDHS risk, and this association was related to BMI after adjusting for smoking and alcohol consumption. At a BMI of 18.5, each additional year of exposure increased UDHS risk by 4.9%. At a BMI of 24, the effect decreased to 2%. Once the BMI reaches 28, the risk of UDHS disappears. The effect of noise exposure duration was near zero when the BMI was greater than or equal to 27.7. According to these results, noise may impair the liver through metabolic and stress compensatory mechanisms, and further clarification of its underlying mechanisms is required based on existing biological evidence.

**Individuals with high BMI exhibit a “saturation effect” in metabolic pathways and a masking of the dominant etiology** In obese individuals, insulin resistance, chronic inflammation, and dysbiosis of the gut microbiota contribute to fatty liver [22–26]. A noisy environment also activates the HPA axis and disrupts metabolic pathways in the gut-liver axis (CREB/CRTC2-PCK1 pathway) [8,18]. In obese individuals with severely dysregulated metabolism, the contribution is significantly reduced, alleviating the detrimental effects. In contrast, lean individuals have relatively better insulin sensitivity, lower baseline inflammation levels, and stable lipid profiles. Metabolic parameters oscillate substantially when exposed to noise stimuli.

**Differences in the “stress elasticity” of the HPA axis** The HPA axis is activated by obesity, affecting metabolism [27]. Nevertheless, this finding implies that long‑term obesity‑related stress may be associated with blunted HPA‑axis responsiveness [28]. Under noise stimulation, it inhibits excessive ACTH and glucocorticoid secretion. Therefore, the adverse effects of excessive glucocorticoid receptor activation are diminished, as is the impairment of liver lipid metabolism.

**Attenuation of the protective effect of peroxisome proliferator-activated receptor alpha (PPAR**α) PPARα is a ligand-activated transcription factor responsible for lipid metabolism, energy homeostasis, and multiple pathophysiological processes. Fat acids and metabolites can activate it [29]. Animal experiments have confirmed that chronic noise exposure and a high-fat diet can exacerbate hepatic steatosis; however, in mice lacking PPARα, the hepatic steatosis is more severe [30].This finding implies that PPARα can, to a certain degree, partially mitigate noise-induced hepatic lipid dysregulation. In people with low BMI, basal fat reserves are inadequate, fatty acid levels are decreased, and PPAR activity is attenuated. Consequently, the effective activation of PPARα-mediated pathways is impaired under noise stress. Conversely, obese individuals sustain chronic high-fat metabolic states and elevated circulating fatty acid levels, allowing the body to establish metabolic compensatory mechanisms before the body experiences the metabolic incident.

### Limitations

This study has several limitations. First, occupational noise exposure was only assessed by exposure duration, without individual noise intensity or cumulative noise dose data. This surrogate measure may cause non‑differential misclassification and bias results toward the null, and no plant‑wide noise data were available for sensitivity analysis. Second, alcohol exposure was defined merely by drinking duration, lacking quantitative information on consumption level and patterns, restricting accurate confounding adjustment. Third, key confounders including physical activity, diet, and occupational physical demands were unavailable, potentially leading to residual confounding. Fourth, the smoking threshold lacked reference support, and we did not distinguish smoking status or calculate cumulative smoking exposure. Future studies incorporating objective personal noise monitoring, quantitative cumulative noise dose assessment, standardized quantitative alcohol consumption evaluation, and comprehensive collection of lifestyle and occupational physical indicators are warranted to minimize exposure misclassification and residual confounding, thereby further validating and refining our findings.

## Conclusions

Exposure to occupational noise demonstrates an independent statistical link to hepatic steatosis, and a substantial interaction exists between noise exposure and BMI within this relationship. Low-weight workers are at high risk of noise-related fatty liver. Given the very small number of female participants, the present findings are limited to male factory workers and may not be extrapolated to females. Occupational health management should formulate differentiated noise protection and metabolic disease prevention strategies based on the BMI levels of workers, to guarantee the liver health of male workers.

## Supporting information

S1 File Supplementary RawData. (XLSX)
